# Unveiling two millennia of ecosystem changes in the Azores through elementome trajectory analysis

**DOI:** 10.1016/j.ecolind.2025.113630

**Published:** 2025-07

**Authors:** J. de la Casa, S. Nogué, M. De Cáceres, S. Pla-Rabés, J. Sardans, M. Benavente, S. Giralt, A. Hernandez, P.M. Raposeiro, J. Peñuelas

**Affiliations:** a Universitat Autònoma de Barcelona, Bellaterra (Cerdanyola del Vallès), Catalonia, Spain; b CREAF, Bellaterra (Cerdanyola del Vallès), Catalonia, Spain; c CSIC, Global Ecology Unit CREAF-CSIC-UAB, 08913 Cerdanyola del Vallès, Catalonia, Spain; d Geosciences Barcelona (GEO3BCN) CSIC, Barcelona, Spain; e GRICA Group, Centro Interdisciplinar de Química e Bioloxía (CICA), Facultade de Ciencias, Universidade da Coruña, Rúa as Carballeiras, 15071 A Coruña, Spain; f CIBIO, Centro de Investigação em Biodiversidade e Recursos Genéticos, InBIO Laboratório Associado, Pólo dos Açores, Ponta Delgada, Portugal; g Faculdade de Ciências e Tecnologia, Universidade dos Açores, Ponta Delgada, Portugal

**Keywords:** Biogeochemistry, Global change, Lake sediments, Trajectory analysis, Paleoecology

## Abstract

The elemental composition of ecosystems responds to environmental perturbations such as land-use change, offering valuable insights into the impacts of global change. This study applies trajectory analysis to two millennia of elemental composition (elementome), including carbon (C), nitrogen (N), and other geochemical elements records from five lakes in the Azores archipelago. By integrating multivariate elemental data, we aim to reconstruct past environmental shifts and understand long-term ecosystem dynamics in response to anthropogenic and non-anthropogenic impacts.

We quantified elementome trajectory analysis metrics, such as elemental turnover, trajectory directionality, and trajectory speed, as ecological indicators to quantify the magnitude and graduality of island ecosystem disturbances. We also tracked elemental shifts over time using CONISS analysis and compared these shifts with previously published environmental reconstructions from the same lakes. Additionally, we analyzed and compared trajectory shapes across the lakes to identify patterns and differences.

Our results indicate that the elementome trajectories primarily reflected changes in erosion rates, in-lake biological activity, and catchment dynamics, characterized by distinct patterns of climate variability, wildfires, and agropastoral activities. Trajectory shapes revealed different patterns across shallow and deeper lakes. Our findings demonstrate the utility of Elementome Trajectory Analysis for disentangling the complex interplay between natural and anthropogenic drivers of ecosystem change in island environments, providing a powerful new tool for paleoecological reconstructions and for monitoring changes at a catchment level.

## Highlights

• The biogeochemical content (elementome) of lake records was analyzed using Elemental Trajectory Analysis.
• Elementome trajectory metrics allowed to assess the magnitude, speed and direction of the observed changes.
• Lake catchment characteristics play a relevant role shaping the elementome trajectories.

## 1 Introduction

Ecological studies often rely on individual elements (e.g. N Cui et al., 2021) or elemental ratios (e.g. C:N:P ratios) (Sardans et al., 2012) to assess resource limitations (Li et al., 2018) or potential stoichiometric imbalances that might cascade through the ecosystem (Peñuelas et al., 2020, Cui et al., 2021). While elemental ratios are undoubtedly relevant for ecosystem analysis, the elementome approach (Peñuelas et al., 2019, Sardans and Peñuelas, 2024) aims to provide a holistic representation of the relative proportion of elements in an ecosystem. The concept of elementome has been used to describe the atomic composition that characterizes an organism or species (Peñuelas et al., 2019, Fernández-Martínez et al., 2021) and hold potential for diverse ecological applications, ranging from understanding ecosystem functioning (Fernández-Martínez 2022) to advancing niche theories (Peñuelas et al., 2019, Fernández-Martínez et al., 2021).

Ecosystem elementomes changes over time. Shifts in these elementomes can be caused by human-related impacts, such as the substantial increase in available C and N due to fossil fuel combustion and extensive fertilization (Sundquist, 1993, Gruber and Galloway, 2008), or by major land use changes associated with agropastoral and mining activities. Similarly, climatic fluctuations and geological events, such as volcanic activity, play significant roles in shaping ecosystemelementomes (Van De Waal et al., 2010, Sun et al., 2021, Rodríguez-Hernández et al., 2022). Given the potential risks of nutrient imbalances and/or stoichiometric disruptions (Peñuelas et al., 2020), enhancing our understanding on elementome shifts and its consequences is important.

Elementome analysis has proven valuable at the organismal level (Peñuelas et al., 2019, Sardans et al., 2021, Fernández-Martínez, 2022), but its application to soils, water bodies, and the whole ecosystems remains limited. This lack of ecosystem-scale data prevents us from fully understanding how shifts in elemental composition drive ecological changes in response to environmental perturbations. Addressing the elemental changes in ecosystems driven by climate, land-use change, and other anthropogenic factors requires an elementome assessment that extends beyond plant elemental composition. Estimating, however, the elementome of an entire ecosystem is a challenging task. Additionally, studying changes in the elementome requires time-series data, especially to understand the consequences of past events such as past climate changes or early anthropogenic impacts. To address these challenges, we propose analysing the elementome within paleoenvironmental records by introducing the concept of the “paleoelementome”.

Lake sediments serve as a natural archive, preserving evidence of past environmental conditions and providing insights into ecosystem responses over time (Williamson et al., 2009, Birks, 2019). They are useful for studying the impacts of contemporary and past environmental change, as they record shifts in ecological and environmental processes (e.g., Catalan et al., 2013, Parducci et al., 2017). Each sediment layer represents a snapshot of the biotic and abiotic processes occurring in the lake, its catchment, and surrounding area at a given time. Various methodologies facilitate the analysis of sediment layers, with fossil pollen providing critical insights into vegetation dynamics (Castilla-Beltrán et al., 2021, Nogué et al., 2022, Nogué et al., 2022) and charcoal particles serving as indicators of fire dynamics (Kulkarni et al., 2021, Sayedi et al., 2024).

Elemental data also play an important role in paleoenvironmental studies by providing complementary information to biological proxies. Traditionally, these studies have focused on geochemical profiles obtained through X-Ray Fluorescence (XRF) scanning, as well as measures of carbon (C), nitrogen (N) alongside stable isotopes analysis conducted using mass spectrometry. These analyses often focused to a subset of elements (e.g. N isotopes indicating changes in the source of N; McLauchlan et al., 2013) or elemental ratios (e.g. Mn/Fe indicating lake oxygenation; Makri et al., 2021). In addition, paleolimnologists use elemental composition to define chemical or biochemical facies within the records (Giralt et al., 2011, Hernández et al., 2017), trace metal pollution (Granmo et al., 2020, Karlsson et al., 2015, Ladwig et al., 2017), and associate the different elements with the mineralogy of the record (Garcia-Oteyza et al., 2024, Giralt et al., 2007, Sánchez-López et al., 2016, Speranza et al., 2019). These diverse methods collectively enhance our understanding of how ecosystems have responded to past environmental changes, providing context for predicting future ecological trajectories, particularly under global change scenarios.

**Fig. 1:**
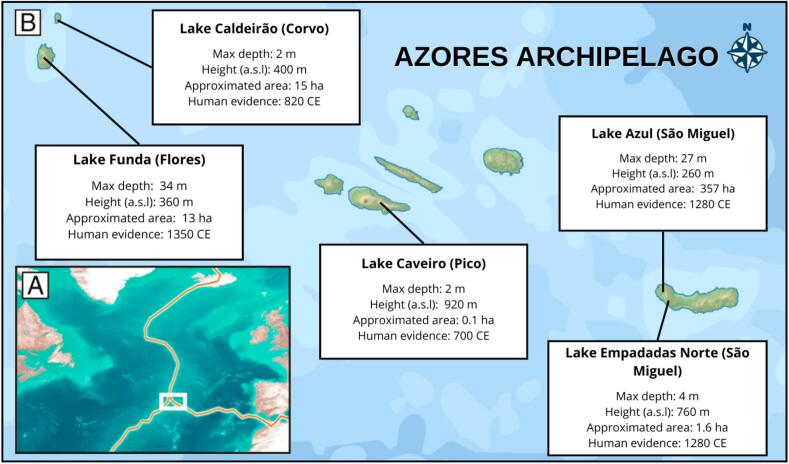
(A) Location of the Azores Archipelago in the North Atlantic. Red lines, triple junction between North American, the Eurasian, and the Nubian plates. (B) Map showing the selected lakes in each island. For each lake, it is indicated the maximum depth (in m), the height above the sea level (in m asl), the approximated area of each lake (in ha) and the year with first evidence of human presence (cal. CE). All information from Raposeiro et al. (2021). (For interpretation of the references to color in this figure legend, the reader is referred to the web version of this article.)

By examining (paleo)elementome trajectories across five distinct lakes from four islands (Flores, Corvo, São Miguel and Pico) in the Azores archipelago (Fig. 1), we aim to characterize significant shifts in elemental composition through trajectory analysis and identify their causes. Our goal is to contextualize paleoelementome dynamics with relevant ecological changes that occurred in each island over the past two millennia, considering both anthopogenic and non-anthropogenic impacts (Hernández et al., 2017, Skov et al., 2010, Raposeiro et al., 2024, Raposeiro et al., 2017, Richter et al., 2022, Ritter et al., 2022, Rull et al., 2017, van Leeuwen et al., 2005, Vázquez-Loureiro et al., 2023).

The trajectory analysis aims to quantify the magnitude, direction, and graduality of the changes in elemental composition over time. While spatial trajectories in two-dimensional space are commonly applied in movement ecology, this concept has recently been extended to multivariate spaces in community ecology (De Cáceres et al., 2019, Sturbois et al., 2023a, Sturbois et al., 2023b, Giguet-Covex et al., 2023, Sánchez-Pinillos et al., 2023, Matthews et al., 2013, Toumi et al., 2023) and has also been applied to other disciplines, such as studies of stable isotopic composition (Sturbois et al., 2021).

We hypothesize that:

1) Trajectory metrics (elemental turnover, directionality, and speed) will indicate the magnitude, graduality, and direction of changes in the elementome. These metrics might reveal shifts that align with human activities and/or climate-related events affecting the lakes and their catchments.
2) Distinct trajectory shapes reflect the characteristics of each lake catchment, including variations in size, depth, and other environmental factors.

## 2 Materials and methods

### 2.1 Study site

The Azores Archipelago is a group of volcanic islands situated between approximately 36°55′N and 39°43′N latitude and 24°46′W and 31°16′W longitude, 1500 km west to mainland Portugal. While the Portuguese claimed to be the first to arrive around 1427 CE recent evidence suggest that the first settlers arrived 700 years before (Raposeiro et al., 2021). The earliest explorers arrived at the end of the early Middle Ages (500900 CE), when temperatures were higher than average, and the westerly winds were weaker, facilitating arrivals to the archipelago from northeastern Europe and inhibiting exploration from southern Europe. This is consistent with archaeological and genetic research suggesting the Norse were the first to colonize the Azores Archipelago. The onset of the Little Ice Age (1300 CE) triggered idoneous climatic conditions for exploration from southern Europe, that might have resulted in the Portuguese colonization of the Azores**.**

### 2.2 Data extraction

We compiled elemental data from five lakes across the Azores archipelago: Lake Caldeirão on Corvo island (39.7023° N, 31.1080° W; Raposeiro et al., 2021); Lake Funda on Flores island (39.4475° N, 31.1939° W, Raposeiro et al., 2021); Lake Caveiro on Pico island (38.43° N, 28.18° W, Benavente 2024) and Lake Empadadas Norte (37.49° N, 25.75° W, Hernández et al., 2017)) and Lake Azul on São Miguel island (37.7804° N, 25.4970° W, Raposeiro et al., 2021); (Fig. 1). Lakes Funda and Azul are the deepest lakes, while lakes Empadadas Norte, Caveiro and Caldeirão are the shallowest (Pereira et al., 2014). Lakes Caldeirão and Caveiro presented the longest records. All lake records cover time-periods before and after the arrival of Portuguese settlers, however, the records of lakes Empadadas Norte and Azul are posterior to the first pre-Portuguese arrival (Raposeiro et al., 2021, Pla-Rabés et al., 2024) (Fig. 1). We selected these five paleoenvironmental records due to the extensive paleoecological research and high-quality elemental data available for them.

The published sedimentary sequences from all lakes included in this study were dated using radiocarbon dating techniques (^14^C and ^210^Pb). We used the published age-depth models, with radiocarbon ages calibrated according to IntCal20 (Reimer et al., 2020; Northern Hemisphere) to assign a calibrated year per each sample (presented in Common Era, CE). The individual sources are Hernández et al., 2017, Raposeiro et al., 2021, Benavente, 2024 and Ritter et al. (2022). For additional methodological details, refer to Raposeiro et al. (2021).

We employed the following elemental compositions: Potassium; K, Silicon; Si, Calcium; Ca, Aluminum; Al, Sulfur; S, Titanium; Ti, Vanadium; V, Chrome; Cr, Manganese; Mn, Iron; Fe; Chlorine; Cl, Strontium; Sr, Bromine; Br and Zirconium; Zr; measured with an Avaatech XRF core scanner. These elements are expressed in counts per second (cps). We also incorporated total organic carbon (C) and total nitrogen (N) measurements, expressed as percentages of dry weight (wt %), into our analysis. We considered organic elements to C and N, and terrigenous elements to K, Si, Al, Mn, Fe, Ti, Zr, V, Sr and Ca. See Raposeiro et al. (2021) for further details on methods.

We complemented the elemental data with available paleoecological data from the same lakes, including fossil pollen percentages of arboreal, shrub, and herbaceous taxa; charcoal particle influx (cm^2^ year^−1^) as an indicator of fire activity; and coprophilous fungi spores influx (cm^2^ year^−1^) as a marker of human and/or cattle presence. For further details, see Raposeiro et al. (2021).

### 2.3 Conceptualization of the elementome and data transformation

All elemental data were first transformed using a square root function to minimize the effect of high variability in elemental records. Some elements were not present across all records due to concentrations under the limit of detection of analytical methods. Thus, individual lake elementomes were constructed using the elements present in each dataset. Subsequently, we applied a min–max transformation, which rescales the values of the minimum value to 0 and the maximum to 1 and allows us to compare between the elements, by standardizing their ranges. This double transformation reduces the weight of highly abundant elements in the dataset (Legendre & Legendre, 2012) very much in the direction of a recent proposed methodology (Bertrand et al., 2024). The values after transformation were interpreted with respect to their relative contents, not by their absolute value.

To understand the relationship between elements, we conducted hierarchical cluster analysis of each element time-series using the Ward method with Euclidean distances (Murtagh and Legendre 2014).

To evaluate the robustness of our approach, we compared elementome analysis under two scenarios: (i) including both XRF and C/N data, and (ii) using XRF data alone. relying only on XRF data. This comparison aimed to assess the method's reliability in studies where only XRF analysis, but not C and N analysis, had been conducted.

### 2.4 Identification of elementome shifts

We first employed stratigraphically constrained cluster analysis of the elementome data, using the stratigraphic constrained incremental sum of squares (CONISS) method (Grim 1987).

Second, we compared our identified paleoelementome zones to the established phases from previous studies. The phases were delignated using multiple proxies, including pollen, spores, charcoal particles, biomarkers, and historical records, providing a detailed reconstruction of human-induced environmental changes over time (Raposeiro et al., 2021). The distinct separation between paleoelementome zones, marked by identified breakpoints in the elemental composition timeline, highlights significant shifts in the elementome.

For Lake Caldeirão and Lake Caveiro, we compared the paleoelementome zones with the four-phase classification of anthropogenic impacts proposed by Raposeiro et al. (2021). Thus, we highlighted the three main breakpoints from the CONISS paleoelementome analysis.

Lake Funda was assessed against the three trophic phases identified by Ritter et al. (2022). Thus, we highlighted the two main breakpoint from the CONISS paleoelemntome analysis.

For Lake Empadadas Norte, five climate-related lake phases were previously established through paleoclimate analysis (Hernández et al., 2017). Thus, we highlighted the four main breakpoint from the CONISS paleoelemntome analysis.

Finally, for Lake Azul, we matched against a three-phase pollen-based phases derived from another sediment core collected in the same lake (Rull et al., 2017). Thus, we highlighted the two main breakpoint from the CONISS paleoelemntome analysis.

### 2.5 Quantification of elementome trajectories

**Fig. 2:**
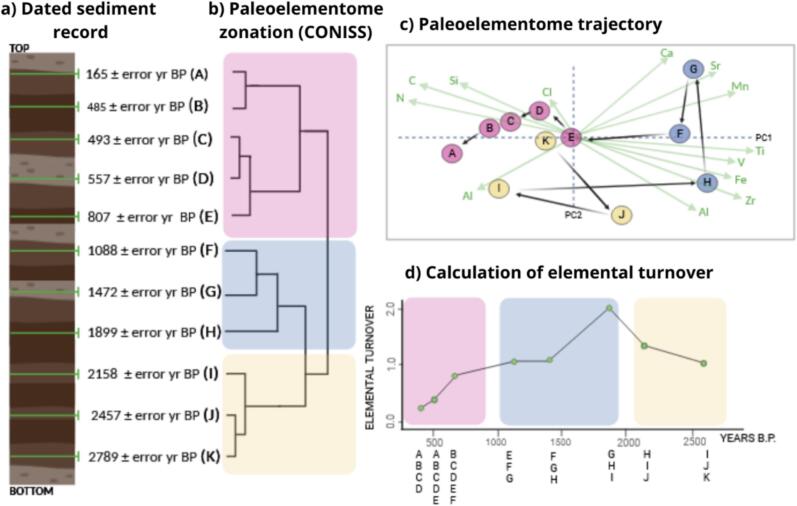
Schematic overview of the trajectory analysis of the paleoelementome based on selected data from the record of Lake Caveiro. (a) Representation of the chronologically-dated sediment core (cal., years BP, Before Present). (b) The clustering of samples based on their elemental composition was represented using stratigraphically constrained CONISS, with different colors indicating distinct zones within the sediment record. (c) Trajectory of the paleoelementome, from oldest to most recent samples, with each sample color-coded according to its CONISS zone. (d) Illustration of the calculation of elemental turnover. The number of samples included in each moving window is stated in the X axis. This figure was created using selected data from the record of Lake Caveiro, as an example we used moving windows defined by the 35 % of the temporal record.

We constructed elementome trajectories to represent the elementome temporal changes of each lake in a multivariate space (Fig. 2). The temporal changes in the elementome can be understood as trajectories in a multivariate space, defined by the elemental composition of the record. Each sample occupies a specific point in the multivariate space, and the trajectories from the position of each consecutive sample can be tracked to obtain information about the shifts in the elementome through time (Giralt et al., 2011).

Principal Component Analysis (PCA) was used to graphically represent the trajectories. To simplify the visualization of the results, we highlight the trajectories between the centroids of the values for each period defined by a stratigraphically constrained CONISS analysis.

First, we estimated the trajectory shapes of each lake, enabling comparison between all of them. We generated a multivariate space using all the data from the five lakes, including only the elements common across the records (C, N, K, Fe, Mn, Ti, Zr, V, Si, Sr, Si, Ca). To describe the trajectory shape, we first calculated the explored area. This metric reflects the amount of the multivariate space explored by each trajectory by calculating the area of the ellipse, that encompassed approximately 95 % of the data points of the record. Ellipses were generated using *stat_ellipse* function from ggplot2 package (Wickham, 2016).

Moreover, to address the similarity between the main clusters (zones) within each record, we calculated another metric, the three-centroid length, that was defined as the sum of the Euclidean distances, in the multivariate space, between the three centroids of the three main clusters of the record, defined by a stratigraphic constrained CONISS. Both trajectory shape metrics: explored area and three-centroid length, were developed for this study.

Secondly, we examined the internal structure of these trajectories by calculating the elemental turnover, directionality and trajectory speed using moving windows. To calculate those metrics, we first defined time-fixed moving windows. The moving windows were defined by a fixed time interval, representing 30 % of total temporal sequence, and applied to each sample, with the year value of the sample as the center of the moving window. The size of the moving window was 581 years for Lake Caldeirão (that spans from 21 to 1986 CE), 533 years for Lake Caveiro (that spans from 7 to 1758 CE), 370 years for Lake Funda (that spans from 774 to 2009 CE), 196 years for Lake Empadadas Norte (that spans from 1353 to 2011 CE) and 194 years for Lake Azul (that spans from 1311 to 1960 CE). The size of the window was chosen to have enough samples (n > 3) represented in each moving window.

After, we calculated the elemental turnover using the beta diversity index by Legendre and de Cáceres (2013) to quantify the variability in elemental composition across time. High values indicate larger differences between samples for the period considered. In combination with moving windows elemental turnover was used to reflect the stability of the paleoelementome across time, expecting low variability values during stable phases and high variability values in disturbance periods. Since sample sizes will not be equally distributed within each defined moving time window, we calculated intervals of confidence of the metric via bootstrap (N = 999) using the R package *boot* (Canty and Ripley, 2024).

For the same moving windows, we also calculated the directionality of the trajectory (De Cáceres et al., 2019) which describes how much a trajectory consistently follows the same direction on the multivariate space, as opposed to non-directional temporal changes. High values of the directionality index indicate that samples have consistently moved towards the same direction for the period considered. We used bootstrapping to generate confidence intervals for elemental turnover, but this was not applicable for directionality, as it is defined as an ordered set of samples.

Finally, to detect the speed of elementome change, we used moving windows and calculated the Euclidean distance between the deepest and shallowest samples within each window, advancing by one sample at a time. We repeated that process for each of the samples between the deeper and shallower sample. Then, we calculated the mean value of all calculations and divided it by the size of the moving window. This was performed for each sample, and the trajectory speed value was assigned to the mean year of the corresponding moving window. Similarly to the directionality of trajectory, bootstrapping was not applicable. To calculate these metrics, we used R (R Core Team 2023) packages, including, *zoo* (Zeileis and Grothendieck, 2005), *codyn* (Hallettet al., 2018), *adespatial* (Dray et al., 2023) *vegan* ((Oksanen et al., 2024) and *ecotraj* (De Cáceres et al., 2019, Sturbois et al., 2023a, Sturbois et al., 2021, Sturbois et al., 2023b).

## 3 Results

### 3.1 Trajectory shapes

We found a similar trend across all lakes. The first axis clearly separated the samples between organic (C and N), and terrigenous elements (K, Si, Al, Mn, Fe, Ti, Zr, V, Sr, Ca, with >70 % variance explained in the first two principal components (Figs. S3-S7, Table S1). We detected differences in the shapes of the trajectories (explored area and three-centroid length) between the lakes depending in their depth and area (Table 1). We found that deeper lakes displayed shorter values of three-centroid length. Lake Azul had the lowest value, followed by Lake Funda, Lake Caldeirão, Lake Empadadas Norte and Lake Caveiro. The explored area value of each trajectory did not show a similar trend (Table 1).

**Table 1:** Lake characteristics and trajectory shapes. The explored area reflects the amount of the multivariate space explored by each trajectory. The three-centroid length addresses the similarity between the three main clusters of the trajectory.

Lake	Area	Maximum depth	Explored area	Tree-centroid length
Azul	357 ha	27 m	0.69	0.07
Funda	13 ha	34 m	0.91	0.16
Caldeirão	15 ha	2 m	0.32	0.19
Empadadas N.	1.6 ha	4 m	1.25	0.36
Caveiro	0.1 ha	2 m	0.59	0.66

### 3.2 Trajectory shifts and metrics

**Fig. 3:**
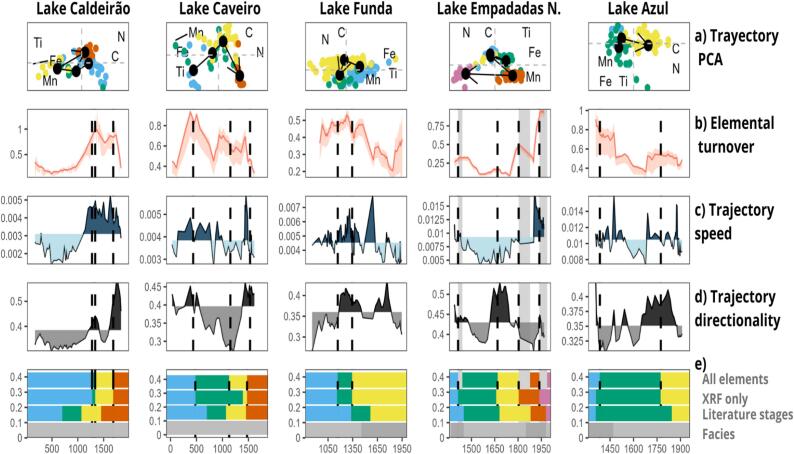
Summary of the Elementome trajectories for the five lakes analyzed in this study. For detailed information of the trajectories of each lake see supplementary information (Figs. S2–S7). In (a) the trajectories within the multivariate space are represented with a PCA. Only C, N, Ti, Fe, and Mn are represented to help the clarity of plot, but the trajectories were constructed using all elements available in each record. The arrow represents the trajectory within the centroids of the different stages identified with stratigraphic constrained CONISS, also depicted by the colors. In (b) the elemental turnover calculated using centered moving windows restricted by time (red line) with confidence intervals calculated with bootstrap (red shade). In (c) the speed of change. Dark blue depicts directionality values above the mean, while light blue depicts values below. In (d) the directionality values. Dark grey depicts directionality values above the mean, while light grey depicts values below. In (e), we compared the three different zones of the paleoecological record. At the top, we show the zones calculated using stratigraphically constrained CONISS with all available elements, followed by those calculated excluding C and N values. At the bottom, the previously identified zones for the same record are presented: the four-phase classification for Caldeirão and Caveiro (proposed in 2021), the three trophic phases for Funda (2022), the six climate-related phases for Empadadas Norte (2017), and the pollen-based phases for Azul (2017). The last bar represents the different sediment facies reported in the study, differenced by shades of grey. Dotted lines across the plots represent shifts in the elementome (identified with CONISS). The dark columns in Empadadas Norte represent data hiatuses. (For interpretation of the references to color in this figure legend, the reader is referred to the web version of this article.)

The results related to elemental turnover, speed and directionality differ between lakes (Fig. 3).

Lakes Caldeirão and Caveiro elementome aligned with some of the previously identified phases of human impacts for Azores (Raposeiro et al., 2021; Fig. 3e). These stages were marked by three boundaries: 700 CE (the arrival of first settlers, which was not synchronic to all islands); 1070 CE (the intensification of human activities); and 1450 CE (the arrival of Portuguese colonizers, coinciding with the onset of the Little Ice Age; LIA). High values of elemental turnover peaks coincide with elementome shifts in both lakes (Fig. 3).

In Lake Caldeirão, any elementome shifts are directly associated with the arrival of Portuguese settlers or the Little Ice Age (LIA). However, around 1500 CE, elemental turnover and trajectory speed were notably high, while directionality remained low, indicating dynamic but non-directional changes in the lake's elemental composition during this period.

In Lake Caveiro, the impact of early settlers on the elementome is characterized by two distinct phases. The initial impact, evident around 900 CE, is marked by high elemental turnover and trajectory speed but very low directionality, signaling the onset of human influence. However, the major elementome shift, driven by intensified agropastoral activities, occurred approximately 430 years later, around 1330 CE. This delayed response suggests that while human presence was detectable early on, significant changes in Lake Caveiro's elemental composition took centuries to manifest, likely due to the gradual intensification of agricultural and pastoral practices (Raposeiro et al., 2021). The Lake Caveiro record has an elementome shift matching with the arrival of Portuguese and the LIA, with high values of directionality and trajectory speed. After the last elementome shift (1685 CE and 1473 CE, for Lakes Caldeirão and Caveiro respectively) in both lakes elemental turnover, directionality, and trajectory speed decreases.

Lake Funda elementome shifts were compared to the Ritter et al. (2022) paleolimnological assessment, that differentiated three distinct trophic stages for the lake and considered a baseline mesotrophic stage from 950 to 1330 CE, a transition stage from 1330 to 1565 CE, and the stabilization of a eutrophic stage from 1565 to 2009 CE. This classification was made considering biomarkers, pollen, spores, diatom assemblages, and sediment geochemistry. The second elementome shift matches with the boundary between the mesotrophic baseline and transition stages, drastically reducing the organic elements and slightly the terrigenous, and associated with agropastoral activities. Elementome turnover, directionality, and trajectory speed were high. No elementome shift matched the stabilization of the eutrophic state of the lake, previously described. Instead, the first elementome shift appears coinciding with the abrupt changes on the vegetation pattern a reduction of trees and shrubs (Ritter et al., 2022) that occurred around 1115 CE.

Lakes Empadadas Norte and Azul are located on the same island, São Miguel, and separated by 5.7 km. The paleoelementome of Lake Empadadas is compared to five previous climate stages assessed by Hernández et al. (2017) that highlighted the climate conditions: first the MCA-LIA transition, a warm and wet phase from 1300 to 1450 CE; secondly, the LIA first half (cold and dry) from 1450 to 1680 CE; third, LIA second half (warm and wet) phase from 1680 to 1880 CE; fourth, the Industrial era, a cold and dry phase from 1880 to 1980 CE (IV) and the current stage, a wet and very hot phase from 1980 to 2011 CE (V). There are three hiatuses in the sediment record from 1414 to 1442 CE; 1804 to 1878 CE and 1937 to 1988 CE. The elementome shifts we identified align closely with the phases proposed by Hernández et al. (2017), likely due to the shared use of biogeochemical proxies. Also, they match with the elemental turnover peaks, except for the second and third elementome shifts, that had low values of elemental turnover and trajectory speed but peaked on directionality. When the climate shifts towards colder and drier conditions, there is an increase in terrigenous elements (e.g., Ti, Zr) due to enhanced erosion and sediment transport from terrestrial sources. At the same time, organic elements (e.g., C and N). The opposite occurs in the transition from cold and dry to wet and hot phases; C and N increase while terrigenous elements decrease.

For Lake Azul, the elementome shifts and the peaks in elemental turnover match with previously described vegetation zones (Rull et al., 2017), but not to the stages by Hernández et al. (2017) that were constructed with the neighbor lake, Empadadas Norte, and should reflect similar climate conditions. The palynological study found three different zones regarding the pollen composition in the sediments. The first pollen zones (1273 to 1358 CE), was dominated by native laurisilva forest. The second zone (1358 to 1845 CE), was characterized by the disappearance of the native laurisilva and the dominance of native shrubs and grass meadows. The last zone (1845 to 2010 CE) was marked by the introduction of commercial tree species and dominance of forestry activities (Rull et al., 2017).

### 3.3 Comparison between different elementome conceptualizations

No differences were observed in the trajectory analysis when comparing an elementome composed of XRF data combined with C and N to an elementome composed solely of XRF data. This indicates that the inclusion of C and N did not significantly alter the trajectory results (Fig. S8). The elementome shifts were similar or slightly similar in most cases, except for the second elementome shift in Lake Caveiro. This shift occurs in the XRF-only approximately 260 years later. In Lake Caveiro, while the trend is maintained, the data range of both elemental turnover and directionality is slightly reduced in the XRF-only approach.

### 3.4 Additional analysis

Finally, an analysis of the trajectories of each lake, focusing on elemental turnover, directionality and trajectory speed, is provided in the Supplementary Information (Figs. S3 – S7). Moreover, to aid in understanding the changes, we provide a detailed plot depicting the individual changes of all elements (including C:N ratio, d^15^N and d^13^C values), in the same timeline for all five lakes (Fig. S1). Additionally, Figs. S9–S13, provide the resulting trees from the cluster analysis of the paleoelementome by stratigraphic constrained CONISS.

## 4 Discussion

### 4.1 Elementome trajectories

It is important to note that the elements originate from diverse sources. Organic elements, particularly C and N, are primarily fixed through biological processes. These elements can originate from the surrounding catchments, atmospheric deposition, or in-lake production processes (Einsele et al., 2001, Harrison et al., 2009). In addition, other elements such as K, Si, Al, Mn, Fe, Ti, Zr, V, Sr, and Ca usually have a lithologic origin. However, Ca and Si can also have a biogenic origin from siliceous and calcareous organisms. Across our records, the second axis of the PCA further differentiated the non-soluble elements, Zr and Sr, from the other terrigenous elements (Figs. S3–S7). This differentiation reflects their behavior as particles that require higher energy for transport to the lake center (Bertrand et al., 2024, Hernández et al., 2017). However, Ti, which is also not soluble does not exhibit the same pattern in the second axis of the PCA. Moreover, we calculated three numerical indicators on elementome trajectories that are already used in community ecology (De Cáceres et al., 2019, Sturbois et al., 2021) but, as far as the authors knows, are novel in the description of biogeochemical data: element turnover, trajectory directionality, and trajectory speed. These metrics provided insights into how lake ecosystems responded to external (e.g. increased runoff in the catchment) and internal disturbances (e.g. shifting algal community and eutrophication). Element turnover quantifies the rate of elemental change, trajectory directionality assesses the consistency of change, and trajectory speed evaluates how quickly the elemental composition changes. By applying these indicators, we can identify periods of rapid or slow change, determine if shifts are consistent or stochastic, and quantify biogeochemical transformations (Table 2)*.*

**Table 2:** Theoretical description of the ecosystem changes through trajectory metrics.

**Elemental turnover**	**Trajectory** **Speed**	**Directionality**	**Interpretation of elementome dynamics**	**Example**
↑	↑	↓	Large and abrupt changes directed towards a new state	Caldeirão third elementome shift. Caveiro first shift.
↑	↓	↑	Large and gradual changes directed towards a new state	Empadadas fourth elementome shift; Azul after first elementome shift
↑	↑	↓	Large and abrupt undirected changes	Any examples found
↑	↓	↓	Large and gradual undirected changes	Caveiro second elementome shift
↓	↓	↑	Smooth and gradual changes towards a new state	Azul second elementome shift
↓	↑	↑	Smooth and abrupt changes directed towards a new state	Funda around 1762 CE
↓	↑	↓	Loose stability (Matthews et al 2013): Smooth and abrupt undirected changes	Funda around 1600 CE
↓	↓	↓	Stabilization	Caldeirão and Caveiro after the last elementome shift

Note: ↑ symbol depicts high or increasing values while ↓ symbol depicts low or decreasing values.

Finally, the combined interpretation of the elementome trajectory metrics shoes periods of stabilization when all metrics (elemental turnover, directionality, and trajectory speed) are low or decreasing. This pattern is particularly clear in Lake Caldeirão after the last major shift around 1650 CE. During this time, land use changed toward pastures in Corvo Island (Raposeiro et al., 2021, Silveira and Dentinho, 2010). The start of this change was marked by an increase in elemental turnover, directionality, and trajectory speed. These findings suggest that land use changes can cause significant shifts in elemental dynamics, followed by stabilization as the system adjusts to new environmental conditions.4.2 Lake catchment influence on elementome dynamics.

A comparison of paleoelementome records from a small and shallow lake (Lake Empadadas Norte) with a large and deep lake (Lake Azul), reveals significant differences in their responses to environmental impacts, despite their proximity of just by 5.7 km (Fig. 1).

The geomorphological characteristics of these lakes clearly influence how their elementomes respond to climatic changes. Lake Empadadas Norte, with a maximum water column depth of four meters, showed a clear pattern. When the climate was wetter and hotter (second elementome shift: 1668–1677 CE; and fourth elementome shift: 1937–1986 CE), the elementome was richer in C and N. Conversely, it was richer in terrigenous elements during colder and drier climatic conditions (first elementome shift: 1414–1442 CE; and third elementome shift: 1804–1878 CE, which also was a period of reforestation). This trend might be explained by an increase in temperature and humidity that could increase the net primary production in the lake and catchment, resulting in an increased accumulation of organic material in the lake sediment (Jiménez-Navarro et al., 2023, Mattsson et al., 2015).

Lake Azul, which has a maximum water column of 27 m, the three paleoelementome metrics increased around 1700 CE. This shift coincides with climate changes in the island during this period, likely reflecting environmental responses to broader climatic shifts such as those associated with the LIA (Hernández et al., 2017). Lake Azul’s sedimentary record has been significantly influenced by volcanic activity and, uniquely among the five lakes studied, is situated within an urbanized area. These factors may confound the interpretation of climate signals by introducing geochemical inputs from volcanism and anthropogenic disturbances, potentially masking their effects on lake productivity and stoichiometry (Vázquez-Loureiro et al., 2019). The comparison of trajectory shapes among the lakes further highlights their geomorphological differences (Benavente 2024). For example, Lake Empadadas, with a three-centroid length value 0.36, displayed more dynamic elementome trajectories compared to Azul (0.07) that is considerably bigger and deep (Table 1, Fig. S2). In addition, lake geomorphology plays an important role driving changes in the elementome, especially with climate. This suggests that small, shallow (and closed) lakes like Lake Empadadas are more sensitive to climatic changes, which tend to induce more pronounced shifts in such environments compared to larger, deeper lakes. (Table 1, Fig. S2).

The differences in trajectory shapes between large and small catchments are influenced by multiple factors. The geomorphology of the catchment defines how the lake is going to expand or shrink with changes in precipitation (Hayashi and van der Kamp, 2007). Moreover, vegetation removal in steep catchments typically enhances erosion rates, as vegetation helps stabilize soils and reduce sediment transport. This process can destabilize steep catchments, leading to increased soil loss and altered geomorphic processes, as observed in studies like Xu et al. (2013). Another limiting factor is the amount of light reaching the bottom of the lake. In shallow lakes, light penetrates the lake bottom, increasing biological activity. In contrast, deep lakes may experience stratification (e.g. Lake Funda; Ritter et al., 2022), leading to anoxic conditions in the bottom. However, this stratification might enhance lake productivity due to P mobilization (Catalan et al., 2024, Cohen, 2003, Tu et al., 2020).

### 4.2 Elementome shifts and anthropogenic ecosystem perturbation

Most of the elementome shifts assessed in this study (via stratigraphic constrained CONISS analysis of the paleoelementome) paired with relevant paleoenvironmental events (Hernández et al., 2017, Rull et al., 2017, Raposeiro et al., 2021, Ritter et al., 2022).

This raises the question of potential temporal mismatches. In Lakes Caldeirão and Caveiro, the elementome shift linked with the impact of first settlers (approximately 700 CE) was delayed by approximately 500 years. However elemental turnover and trajectory speed were increasing since the arrival of first settlers, indicating an accumulation of anthropogenic impacts from the arrival of first settlers until the elementome shift occurred (Fig. 3). Additional temporal mismatches between our observed elemental shifts and the previously established phases are evident in Lakes Caveiro and Funda. The first elementome shift in Lake Caveiro occurred before the arrival of the first settlers and coincided with a decline in tree species (e.g. mainly *Juniperus brevifolia*). This change is attributed to volcanic activity (Connor et al., 2012. In Lake Funda, only one elementome shift (the second: 1168–1178 CE) matches with a previously stated trophic stage (Ritter et al., 2022). The first elementome shift occurs between 1168–1178 CE, with a peak in elemental turnover and without a direct correspondence in previously identified phases (Ritter et al., 2022). The observed shift coincides with changes in multiple paleoenvironmental proxies. For example, Raposeiro et al. (2021) reported on Lake Funda a sudden decrease followed by an increase in arboreal pollen, suggesting significant vegetation changes. Concurrently, Ritter et al. (2022) documented shifts in *Chironomidae* communities and diatom assemblages, indicating an increase in lake productivity.

Human impact in our records could be associated mainly with an increase in the fire regime and/or changes on land-use change (Nogué et al 2017). Fire regimes played a role in influencing elementome shifts in Lake Caldeirão (first elementome shift: 1275–1278 CE), Caveiro (second elementome shift: 1129–1143 CE), Funda (1100–1200 CE) and Azul (first elementome shift: 1385–1391 CE and second: 1774–1799 CE) (Figs. S1–S7). These shifts were shaped by variations in fire frequency, intensity, and seasonality, which impacted nutrient cycling and sediment composition (Raposeiro et al., 2021). Fire events in the lakes Caldeirão and Caveiro were characterized by a high elemental turnover and speed. Directionality was variable. This may be caused by a combination of increased erosion due to land degradation, leading to prevalence of terrigenous elements such as Ti or Zr, along with the input of charcoal ashes and wood debris, which can have high concentrations of C or other elements as K or Si (Smołka-Danielowska and Jabłońska, 2021).

Land use change is a major driver of change when humans arrive on islands (Nogué et al., 2017, Nogué et al., 2024). While we do not have historical information prior to Portuguese colonization, later historical documents provide evidence of forest burnings, cereal cultivation, and animal husbandry on the islands following the arrival of the Portuguese (Frutuoso, 1981, Moreira, 1987). The introduction of pastures is one of the most relevant land use changes in the environmental records of islands (Walsh et al., 2020). In our analysis, land use changes are typically associated with increases in carbon (C) and nitrogen (N) concentrations (Hernández et al., 2013, Ritter et al., 2022). These elevated levels can be attributed to two primary sources: enhanced inputs from the catchment area or increased in-lake production. The former may result from soil disturbance and erosion following land conversion, while the latter could be a consequence of altered nutrient dynamics stimulating aquatic primary productivity. For example, the introduction of pastures in Lake Caldeirão increased trajectory speed, directionality, and elemental turnover, until a point (the third elementome shift: 1685–1715 CE) after they started decreasing, indicating certain stabilization (Fig. 3). This more stable period conicides with the onset of the dominance of herbaceous taxa in the pollen composition (Fig. S3) that represents the stabilization of the pasture ecosystem (Raposeiro et al. 2021). In Lake Caveiro, the introduction of agropastoral activities led to an increase in directionality values, that was low, followed by an increase in trajectory speed and elemental turnover, which all peaked before the third elementome shift (year 1100 CE). After that, elemental turnover and trajectory speed decrease while directionality increases, smoothly shifting towards a more dominated C and N elementome (Fig. S4).

Around 1350 CE, grazing activities in Lake Funda coincided with the second elementome shift, marked by a peak in all three metrics (elemental turnover, trajectory directionality, and speed). This shift, however, resulted in a reduction of both organic and terrigenous elements. The pollen diagram for Lake Funda indicates that agropastoral activities led to a shrub-dominated landscape (e.g., *Myrsine africana*) with a decline in tree cover (Fig. S5). This contrasts with Lake Caldeirão, where the shrub ratio remained stable over time (Fig. S3). The replacement of forests by shrubs and pastures, without an increase in fire regimes, suggests logging activities as the primary driver. This could explain why, despite agropastoral land use changes, the organic element signal did not increase.

### 4.3 Limitations and perspectives

Our study simplifies complex data on sedimentary elemental composition into quantitative metrics. These metrics facilitate tracking environmental changes over a span of two millennia. We believe this approach has potential to reveal how elemental dynamics in ecosystems have changed over time. However, we also understand that lake sediment records mainly reflect sedimentary processes. To get a fuller picture of how ecosystems change, future research should also include other natural archives like soil layers, peat deposits, or long-lived trees. These could help uncover changes in both the biotic and abiotic components of ecosystems. In this study, we constructed an elementome using all available elements and the C/N ratio. However, we acknowledge that some paleoenvironmental studies rely solely on XRF data, excluding C and N measurements. To address this limitation, we conducted a parallel analysis using only XRF-measured elements, which yielded similar results (Fig. S8). This suggests that our approach is robust even when C and N data are unavailable. It is important to note that we do not recommend substituting C and N measurements with loss on ignition (LOI) values. While LOI is sometimes used as a proxy for organic matter content, it can introduce bias in carbon content estimates, as demonstrated by Santisteban et al. (2004). This potential for inaccuracy underscores the importance of direct C and N measurements when possible.

While in this study we explored four metrics of elementome trajectories, there are additional possibilities that may be more suitable for other research questions. One metric that could also be explored is the trajectory distance toward a reference point (Sturbois et al., 2021). This metric is potentially useful for ecosystem restoration purposes with a clear restoration baseline or when research questions focus on specific and detailed impacts. Additionally, working with smoothed trajectories from the package *ecotraj* (De Cáceres et al., 2019, Sturbois et al., 2021, 2023), could help when dealing with noisy datasets from lake records.

Together, elemental turnover, directionality, and trajectory speed characterized the evolution of the ecosystem elementome through time and served as ecological indicators of the environmental changes in the lake and catchment. While we have proven its potential in paleoenvironmental records, the scope of elementome trajectory analysis extends beyond. Elemental trajectory analysis demonstrates significant potential as a valuable tool for ecologists and paleolimnologists to monitor the stability of elementomes in ecosystems or organisms, and to provide insights into biogeochemical dynamics over time.

## 5 Conclusion

Through the lens of “atomical ecology” (Sardans & Peñuelas 2024), we introduced and tested trajectory analysis for examining elemental data from sedimentary sequences, termed elementome trajectory analysis. This approach involved the trajectories of different samples within a multivariate space constructed from the elemental composition of the records. By utilizing this method, we were able to track shifts in ecosystem elementomes, thereby inferring the link with different ecosystem perturbations. For example, C and N played a role when biological activity within the catchment or lake increases. In contrast, terrigenous elements (K, Si, Al, Mn, Fe, Ti, Zr, V, Sr, and Ca) reflect increased runoff and erosion, a pattern observableacross all studied systems.

Our study demonstrates the effectiveness of this approach in revealing how ecosystems have responded to long-term environmental changes. Furthermore, we introduced trajectory metrics for interpreting elemental time series: elemental turnover, trajectory speed, and directionality. These metrics serve as proxies for changes in magnitude, graduality, and directionality of the changes occurring in the lake and catchment. We described and compared trajectory shapes by quantifying the multivariate space explored for each trajectory (explored area) and the distance between the major clusters in the record (three-centroid length). The three-centroid length is directly related to catchment area, indicating that small, shallow lakes experience larger shifts than larger, deeper lakes.

Finally we would like to encourage the use of elementome trajectory analysis in environmental reconstructions to optimize the potential of elemental databases, enhance the depth of interpretations, and assess the magnitude, graduality, and direction of the observed changes. Additionally, this method serves as a powerful tool to monitor and untangle the intricate relationships between the elemental structure of ecosystems and the fundamental processes driving natural systems.

## CRediT authorship contribution statement

**J. de la Casa:** Writing – original draft, Methodology, Investigation, Formal analysis, Data curation, Conceptualization. **S. Nogué:** Writing – review & editing, Supervision, Data curation, Conceptualization. **M. De Cáceres:** Writing – review & editing, Methodology, Conceptualization. **S. Pla-Rabés:** Writing – review & editing, Methodology. **J. Sardans:** Writing – review & editing, Supervision. **M. Benavente:** Writing – review & editing, Data curation. **S. Giralt:** Writing – review & editing, Data curation. **A. Hernandez:** Writing – review & editing, Data curation. **P.M. Raposeiro:** Writing – review & editing, Data curation. **J. Peñuelas:** Writing – review & editing, Supervision, Conceptualization.

## Declaration of competing interest

The authors declare that they have no known competing financial interests or personal relationships that could have appeared to influence the work reported in this paper.

## Data Availability

Each lake record is available from Hernández et al., 2017, Raposeiro et al., 2021, Benavente, 2024 and Ritter et al. (2022). The exact database used for the study is available upon request.
