# Supplementary material for: Unveiling two millennia of ecosystem changes in the Azores through elementome trajectory analysis
Source: Ecol Indic. 2025 Jul;176:113630. doi: 10.1016/j.ecolind.2025.113630 (PMC12170352; doi:10.1016/j.ecolind.2025.113630)
Supplement: Supplementary Data 1 [file mmc1.docx]

Supplementary information for:

**Unveiling two millennia of ecosystem changes in the Azores through Elementome Trajectory Analysis**

**This pdf includes:**

**Single element trends in all lake records**

**Figure S1** – Page 2

**Comparison of the trajectories in the same multivariate space**

**Figure S2** – Page 3

**Detailed trajectory analysis of each lake**

**Figure S3 (Caldeirao)** – Page 4

**Figure S4 (Caveiro)** – Page 5

**Figure S5 (Funda)** – Page 6

**Figure S6 (Empadadas)** – Page 7

**Figure S7 (Azul)** – Page 8

**Comparison of trajectory metrics including or excluding C and N**

**Figure S8** – Page9

**CONISS dendrograms for each lake**

**Figure S9 (Caldeirao)** – Page 10

**Figure S10 (Caveiro)** – Page 11

**Figure S11 (Funda)** – Page 12

**Figure S12 (Empadadas)** – Page 13

**Figure S13 (Azul)** – Page 14


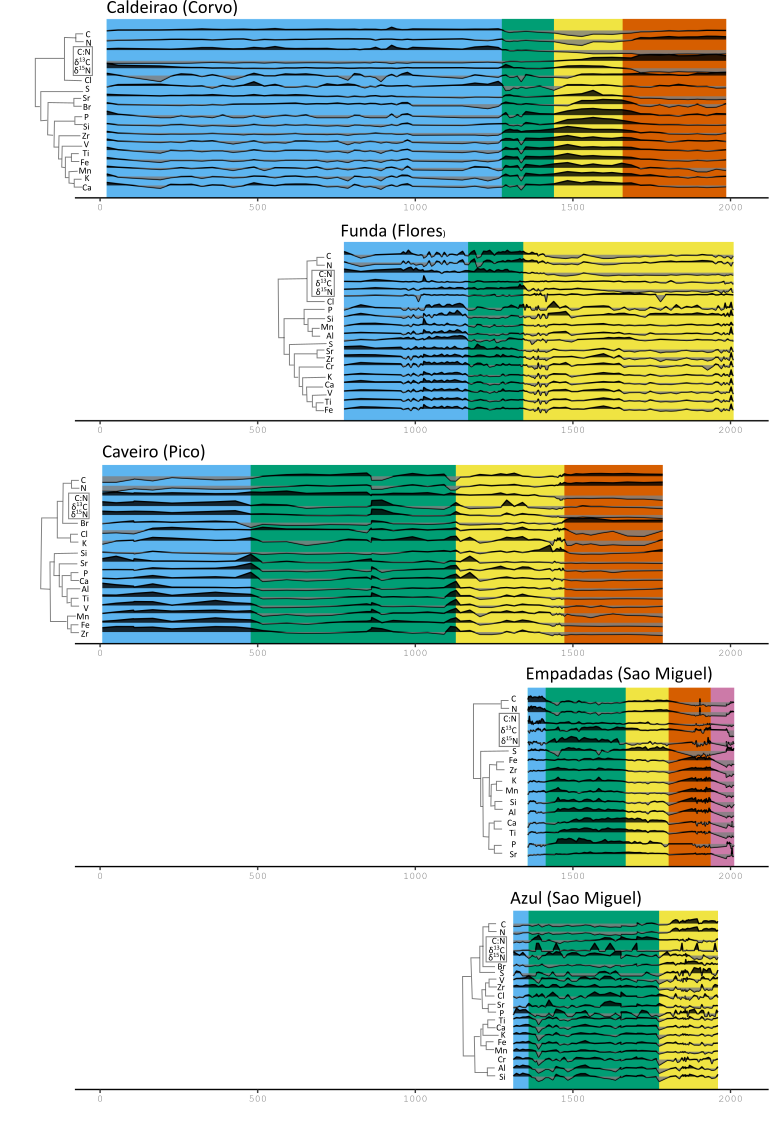


**Figure S1**. Evolution of the relative content of each element for the five lakes. The values of element are standardized data. Although P was rejected for the trajectory analysis due to low resolution with the XRF analysis, we include the data here for anyone interested.


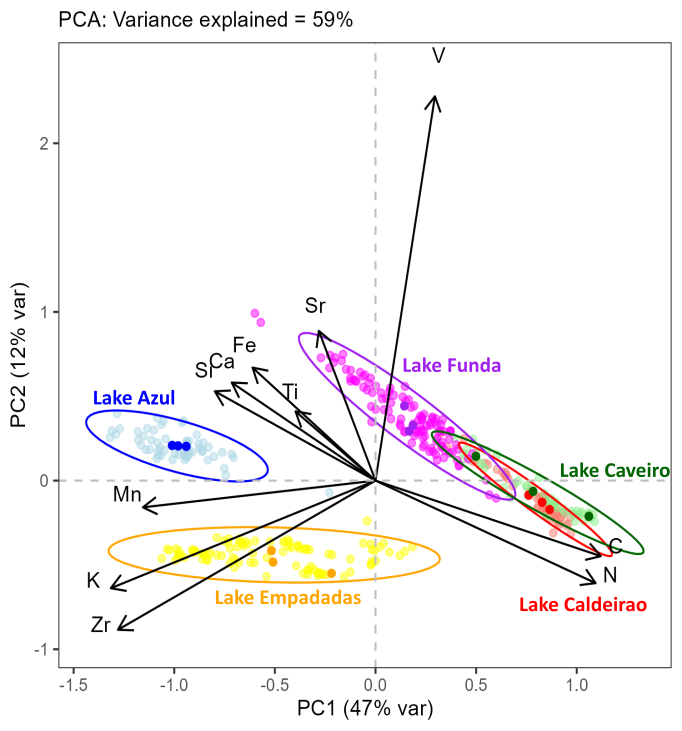


**Figure S2**. Trajectories of the five lakes in a shared multivariate space. Darker dots represent the three main centroids of each record. Three-centroid length was calculated as the sum of the lengths between the centroids. The ellipse represents the area occupied by the 95% of the samples. The explored area was calculated using the area of the ellipses.

**
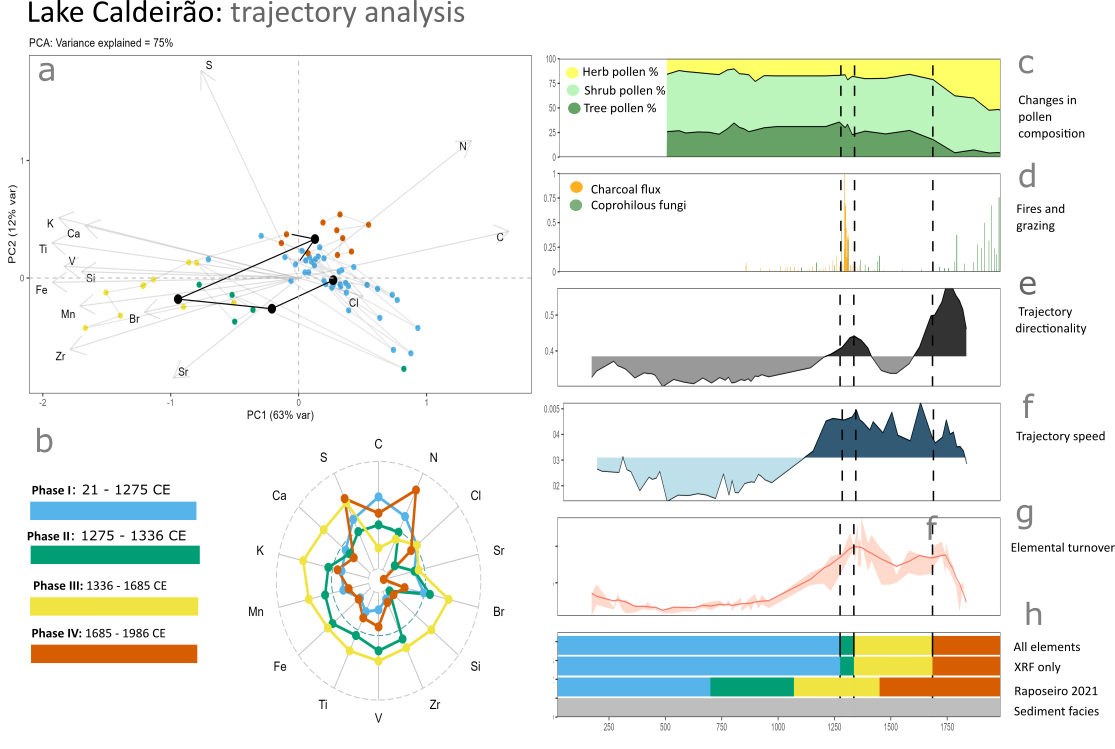
**

**Figure S3.** *Lake Caldeirão paleoelementome and palynology analysis. (a) is the paleoelementome trajectory represented in a PCA plot. (b) is a spider plot of the characterization of the elementome during the different zones. The elements are ordered following the Ward analysis (Figure S1) (c) is the change in pollen composition of herb (yellow), shrub (light green) and tree (dark green) species, in % (axis from 0 to 100%). (d) indicates the fire regimen (orange) by the charcoal flux and the cattle presence (green) by coprophilus fungi spore flux (rescaled values from 0 to 1). (e) is the trajectory directionality. Dark gray depicts values over the mean of the trend; while light gray indicates values under the mean. (f) is the trajectory speed. Dark blue depicts values over the mean of the trend; while light blue indicates values under the mean. (g) is the elemental turnover and its confidence interval generated via bootstrap. In (h) are the different temporal zones constructed with the paleoelementome (all elements, and XRF only), and the ones previously determined by Raposeiro (ref) taking into account the anthropogenic stages of the lake. The colors of a, b and e (only the upper zonation) correspond to the paleoelementome zonations: blue for the first (500 – 1275 CE), green for the second (1275– 1336 CE), yellow for the third (1360 - 1685), and orange for the last (1685– 1937 CE). On gray, the sedimentary facies. In this case it only presented one facies: reddish brown mud. The dotted line in c-g represent the elementome shift (the oldest date from the interval)*

*
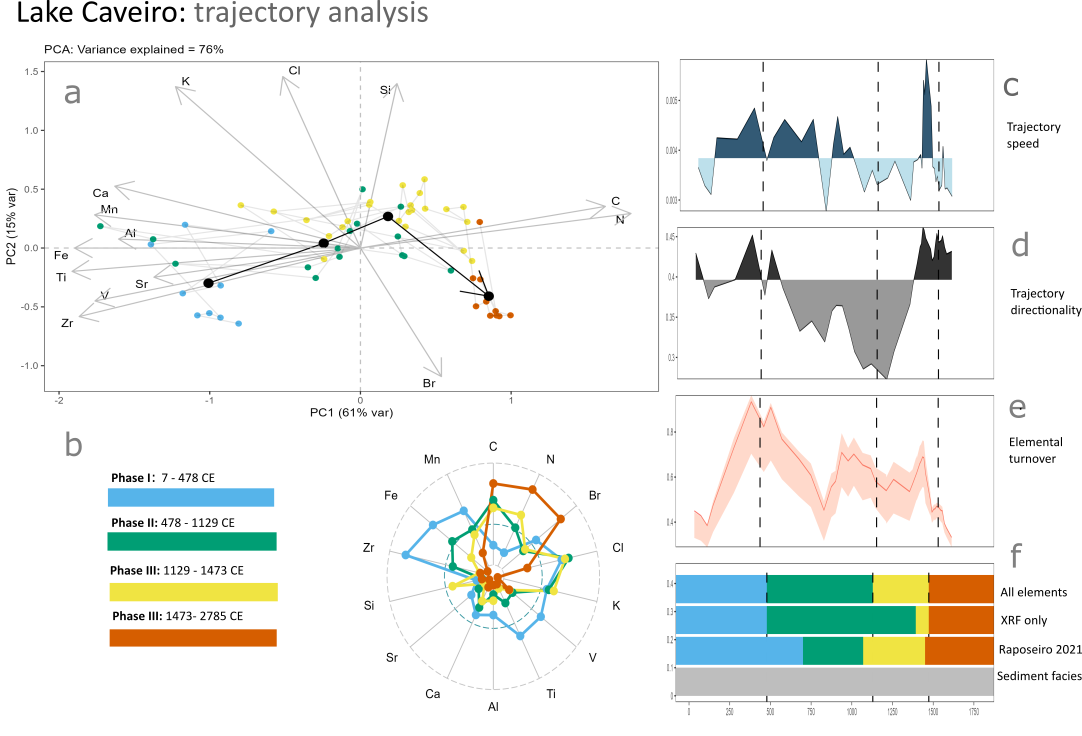
*

**Figure S4.** *Lake Caveiro paleoelementome analysis. (a) is the paleoelementome trajectory represented in a PCA plot. (b) is a spider plot of the characterization of the elementome during the different zones. The dots over the element name indicate a significant difference between this zone (the one of the color) and the previous. The elements are ordered following the Ward analysis (Figure S1). (c) is the trajectory speed. Dark blue depicts values over the mean of the trend; while light blue indicates values under the mean (d) is the trajectory directionality. Dark blue depicts values over the mean of the trend; while light blue indicates values under the mean. Dark grey depicts values over the mean of the trend; while light grey indicates values under the mean. (e) is the elemental turnover with a confidence interval generated via bootstrap. In (f) are the different temporal zones constructed with the paleoelementome (all elements, and XRF only), and the ones previously determined by Raposeiro (ref) taking into account the anthropogenic stages of the lake. The colors of a, b and e (only the upper zonation) correspond to the paleoelementome zonations: blue for the first (7 – 478 CE), green for the second (478 – 1129 CE), yellow for the third (1129 – 1434 CE) and orange for the last (1434 – 1785 CE). For this record, we didn’t set a ceal sediment facies classification. The dotted line in c-e represent the elementome shift (the oldest date from the interval)*

*
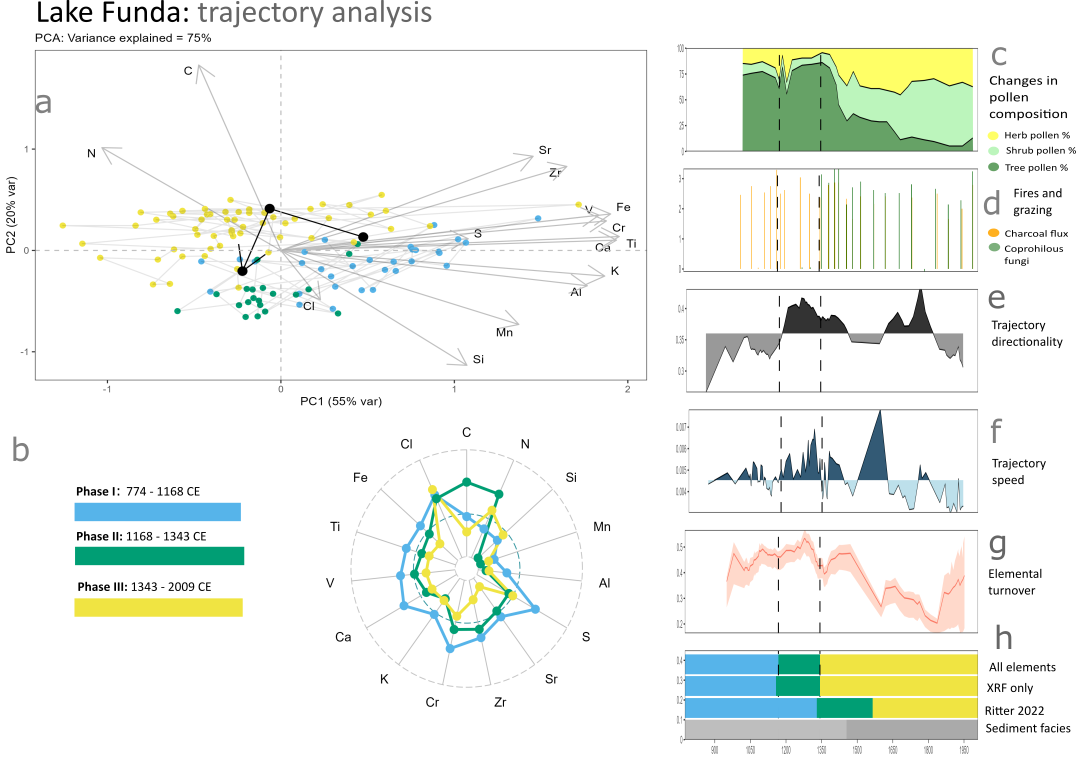
*

**Figure S5.** *Lake Funda paleoelementome and palynology analysis. (a) is the paleoelementome trajectory represented in a PCA plot. (b) is a spider plot of the characterization of the elementome during the different zones. The dots over the element name indicates significant difference between this zone (the one of the colour) and the previous. The elements are ordered following the Ward analysis (Figure S1). (c) is the change in pollen composition of herb (yellow), shrub (light green) and tree (dark green) species, in % (axis from 0 to 100%). (d) indicates the fire regimen (orange) by the charcoal flux and the cattle presence (green) by coprophilous fungi spore flux (rescaled values from 0 to 1). ). (e) is the trajectory directionality. Dark gray depicts values over the mean of the trend; while light gray indicates values under the mean. (f) is the trajectory speed. Dark blue depicts values over the mean of the trend; while light blue indicates values under the mean. (g) is the elemental turnover and its confidence interval generated via bootstrap. In (h) are the different temporal zones constructed with the paleoelementome (all elements, and XRF only), and the ones previously determined by Ritter (ref) taking into account the lake trophic stages. The colors of a, b and e (only the upper zonation) correspond to the paleoelementome zonations:* green for the first (776 – 1668 CE), yellow for the second (1668 – 1434 CE) and orange for the last (1434 – 2009 CE). In gray/dark gray the different facies: first one (776 – 1455 CE) mud, sand and gravels; the second (1455 – 2009 CE) laminated deposit. *The dotted line in c-g represent the elementome shift (the oldest date from the interval)*


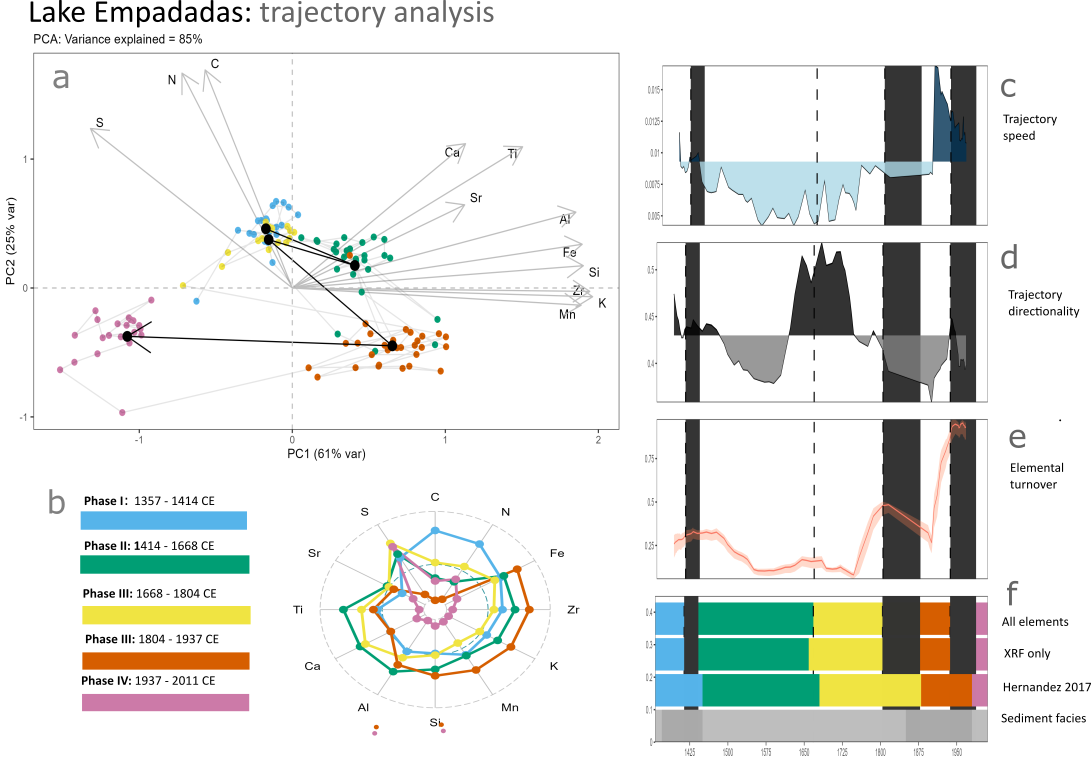


**Figure S6.**  *Lake Empadadas paleoelementome analysis. (a) is the paleoelementome trajectory represented in a PCA plot. (b) is a spider plot of the characterization of the elementome during the different zones. The dots over the element name indicate a significant difference between this zone (the one of the color) and the previous. The elements are ordered following the Ward analysis (Figure S1). (c) is the trajectory speed. Dark blue depicts values over the mean of the trend; while light blue indicates values under the mean (d) is the trajectory directionality. Dark blue depicts values over the mean of the trend; while light blue indicates values under the mean. Dark grey depicts values over the mean of the trend; while light grey indicates values under the mean. (e) is the elemental turnover with a confidence interval generated via bootstrap. In (f) are the different temporal zones constructed with the paleoelementome (all elements, and XRF only), and the ones previously determined by Hernández et al. 2017 (ref) taking into account the climate. The colors of a, b and c (only the upper zonation) correspond to the paleoelementome zonations: blue for the first (1357 – 1414 CE), green for the second (1414– 1668CE), yellow for the third (1668 – 1804 CE), orange for the fourth (1878 – 1937 CE) and pink for the last (1988 – 2011 CE). In gray/dark gray the different facies: first one (until 1350 CE) volcanic clastic deposits; the second (1450 – 1450 CE) dark-brown muddy sediments; third one (1450-1850 CE) banned brown mud and silt, fourth one (1850 – 1980 CE) dark to pale brown silts, with a sand band at 1890 CE; and fifth one (1980 – 2011 CE) brown to ochre mud very rich in diatoms. Black boxes depict hiatus. The dotted line in c-e represent the elementome shift (the oldest date from the interval)*


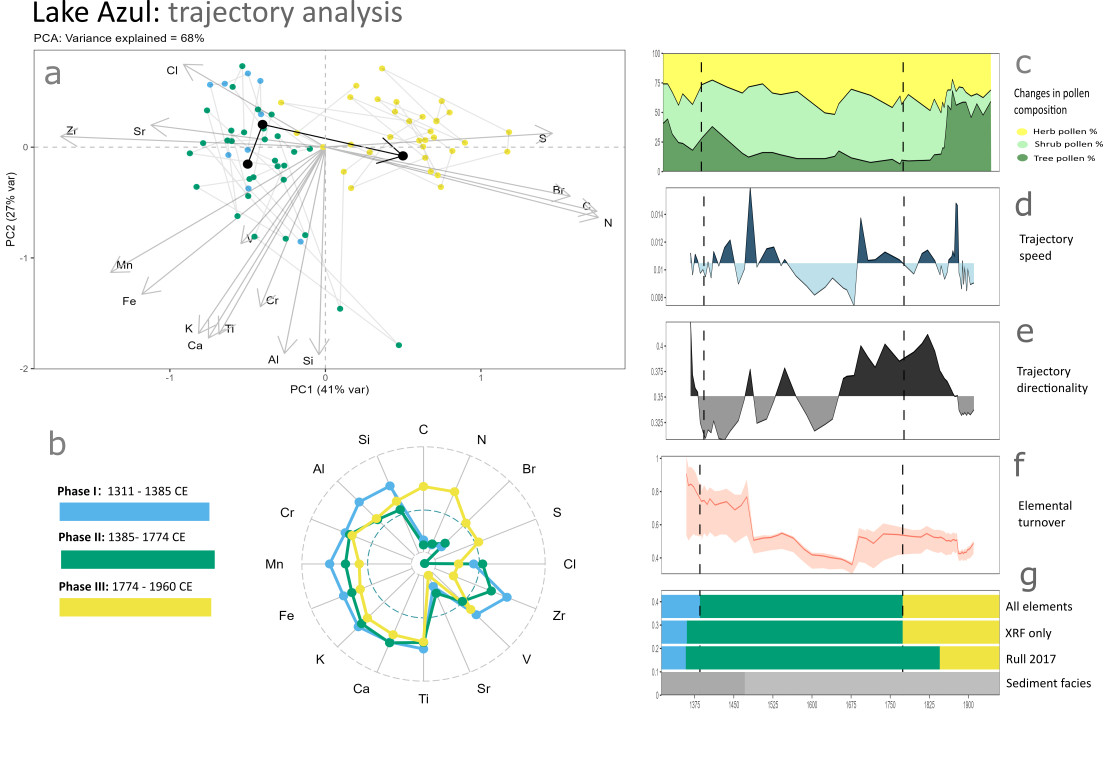


**Figure S7.** *Lake Azul paleoelementome and palynology analysis. (a) is the paleoelementome trajectory represented in a PCA plot. (b) is a spider plot of the characterization of the elementome during the different zones. The dots over the element name indicate a significant difference between this zone (the one of the color) and the previous. The elements are ordered following the Ward analysis (Figure S1). (c) is the change in pollen composition of herb (yellow), shrub (light green) and tree (dark green) species, in % (axis from 0 to 100%) This data is not from the same core, but from a previous publication (Rull et al 2017 ref). (d) is the trajectory speed. Dark blue depicts values over the mean of the trend; while light blue indicates values under the mean (e) is the trajectory directionality. Dark blue depicts values over the mean of the trend; while light blue indicates values under the mean. Dark grey depicts values over the mean of the trend; while light grey indicates values under the mean. (f) is the elemental turnover with a confidence interval generated via bootstrap In (g) are the different temporal zones constructed with the paleoelementome (all elements, and XRF only), and the ones previously determined by Rull (ref) taking into account the pollen record. The colors of a, b and d (only the upper zonation) correspond to the paleoelementome zonations: blue for the first (1331 - 1385 CE), green for the second (1385 – 1774 CE) and yellow for the last (1774 – 1960 CE). In gray/dark gray the different facies: first one (1331 – 1471 CE) gray to brown laminated silty mud; the second (1471 – 1960 CE) is brown mud. The dotted line in c-f represent the elementome shift (the oldest date from the interval)*


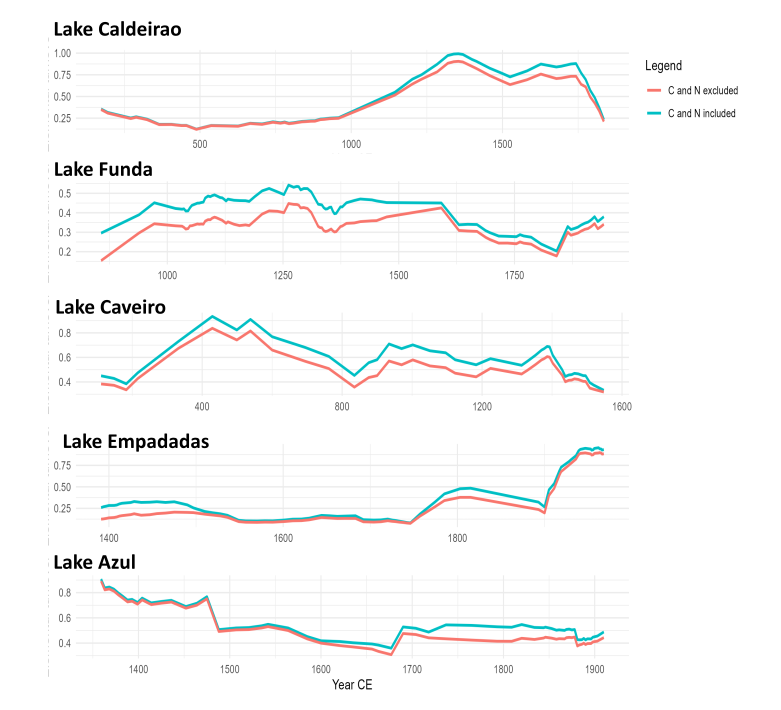


**Figure S8.**  *Comparison of elemental turnover with and without excluding C and N from the analysis. Blue lines represent C and N included, while red lines represent C and N excluded. Most of the elementome shifts were similar in both approaches. The general trends for elemental turnover and directionality were also similar (Figure S3-8). The most relevant difference is in the second elementome shift of Caveiro (1129-1143 CE), that occurred around 260 years later in the XRF-only elementome. This evidence the strong opposite correlation between organic and terrigenous elements. Even without the data for the C and N, the trajectories of the XRF-only paleoelementome are alike because instead of going to the C and N direction in the PCA, they are following the opposite direction to the terrigenous signal. However, we encourage the inclusion of C and N when building elementomes as they provide key information for the ecosystems. The trajectory analysis of Lake Empanadas is one example. After the third shift in the complete elementome approach (1804-1878 CE; Fig S8), there is a reduction of both terrigenous and organic elements. This is not evident when looking only at the XRF elementome. In this case for XRF-only elementomes, this could be interpreted as an increase on the organic proportion of the paleoelementome. Also, the oscillation between the first (1414-1442 CE) and second (1668-1677 CE) elementome shifts is not present when C and N are not considered*


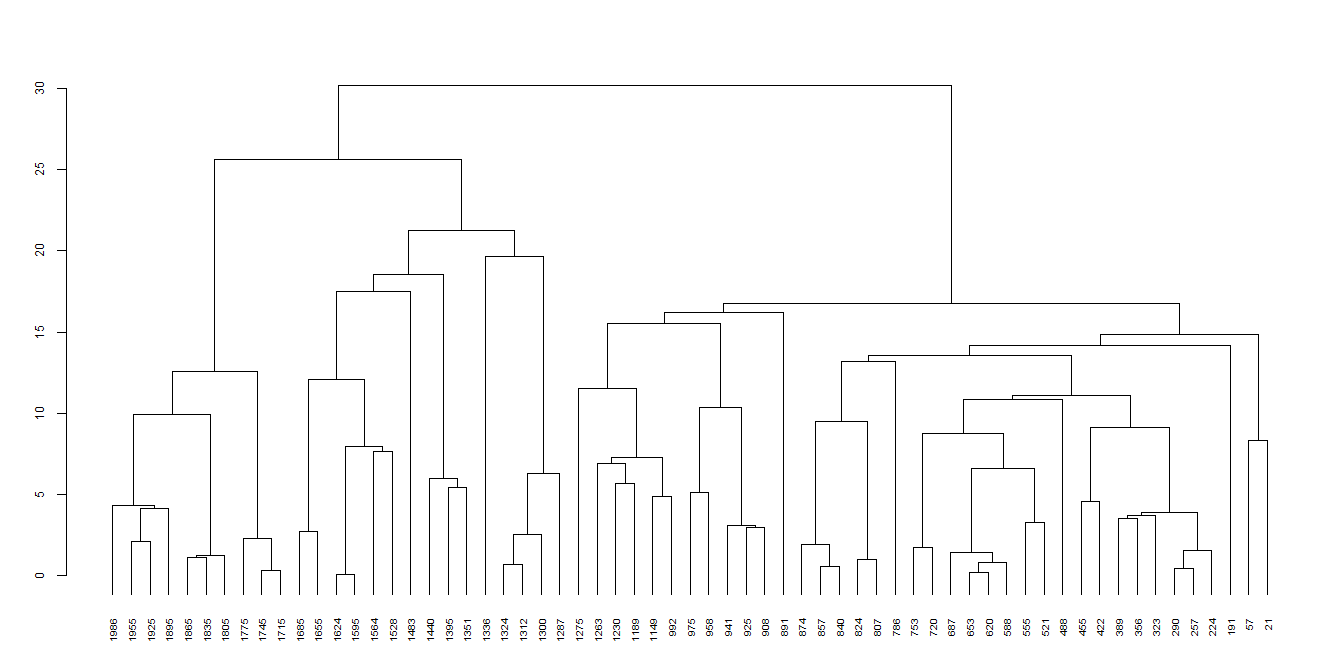


**Figure S9**. Dendrogram of the CONISS analysis of the paleoelementome of Lake Caldeirao.


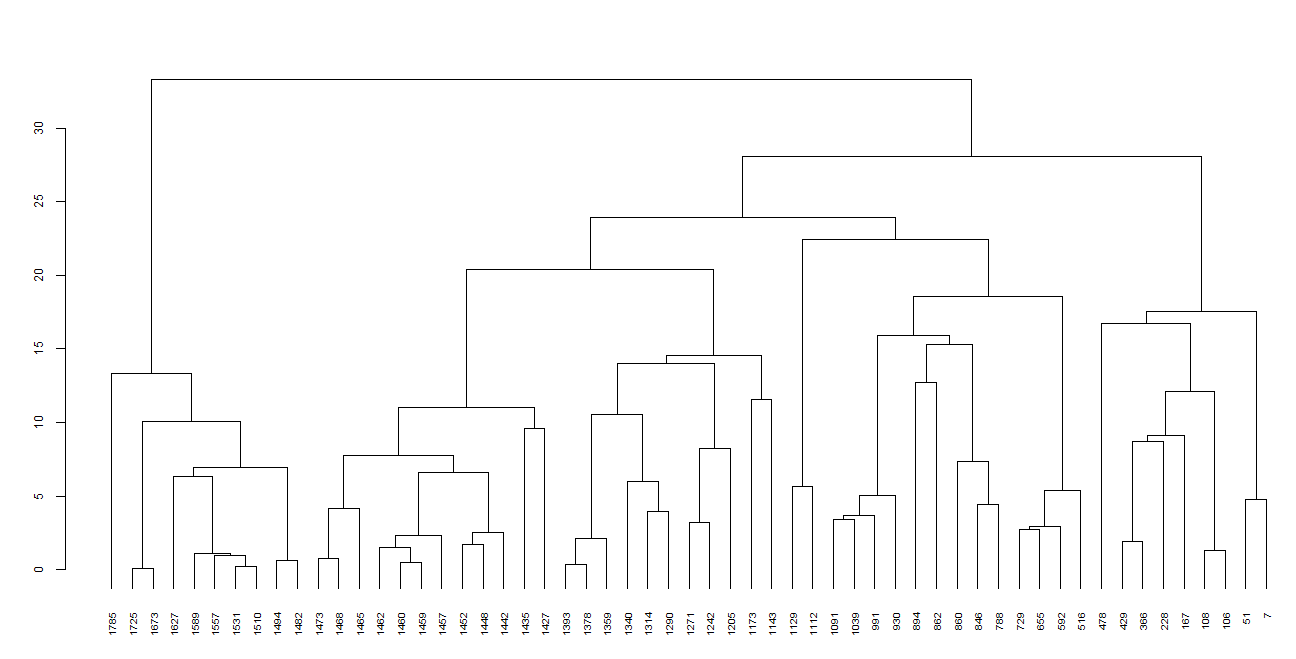


**Figure S10**. Dendrogram of the CONISS analysis of the paleoelementome of Lake Caveiro.


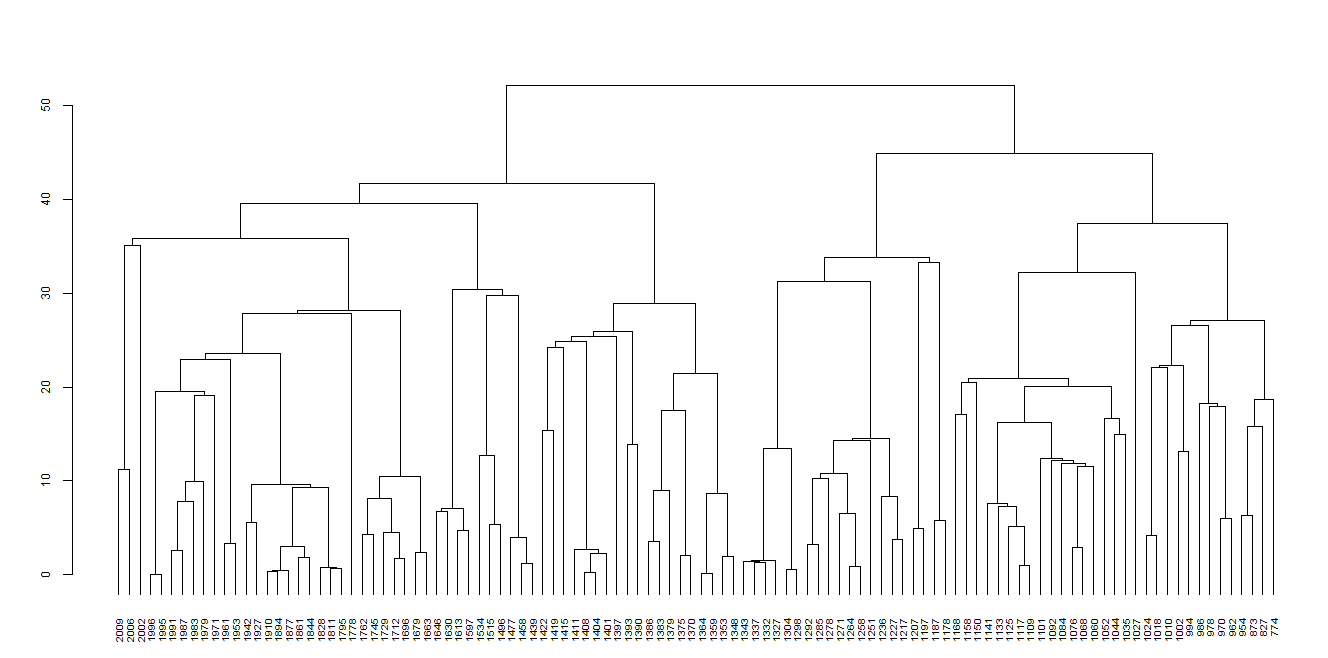


**Figure S11**. Dendrogram of the CONISS analysis of the paleoelementome of Lake Funda.


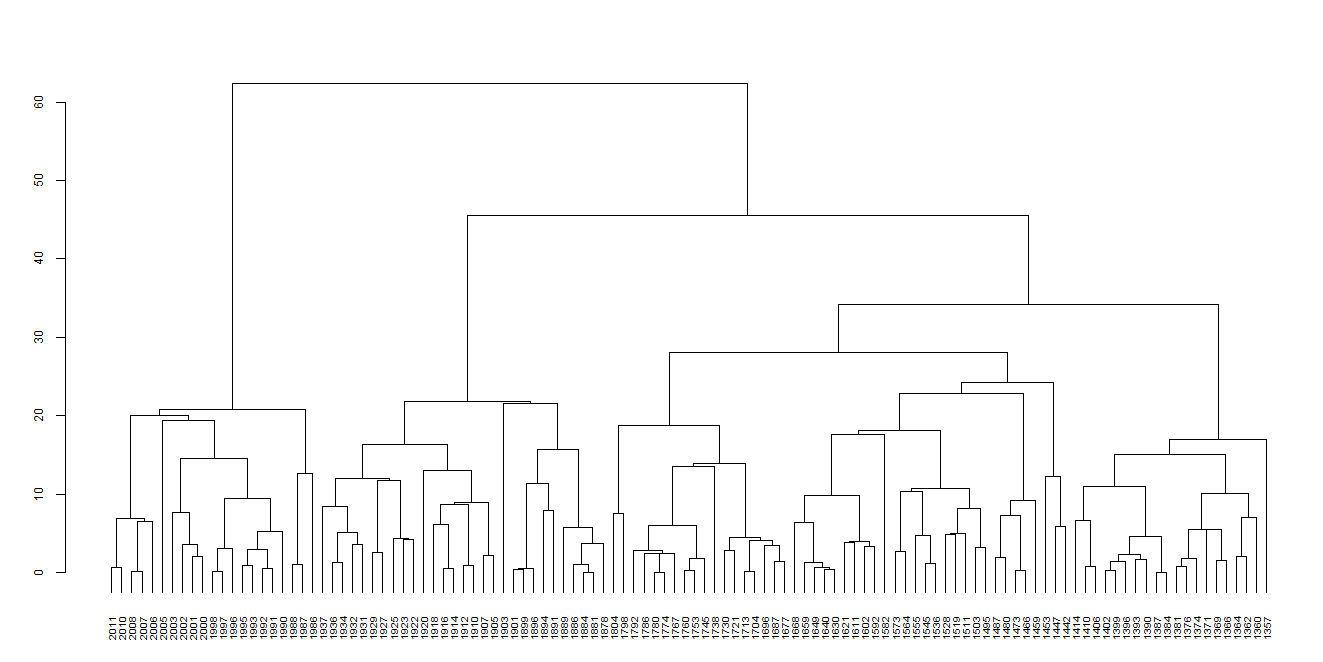


**Figure S12**. Dendrogram of the CONISS analysis of the paleoelementome of Lake Empadadas.


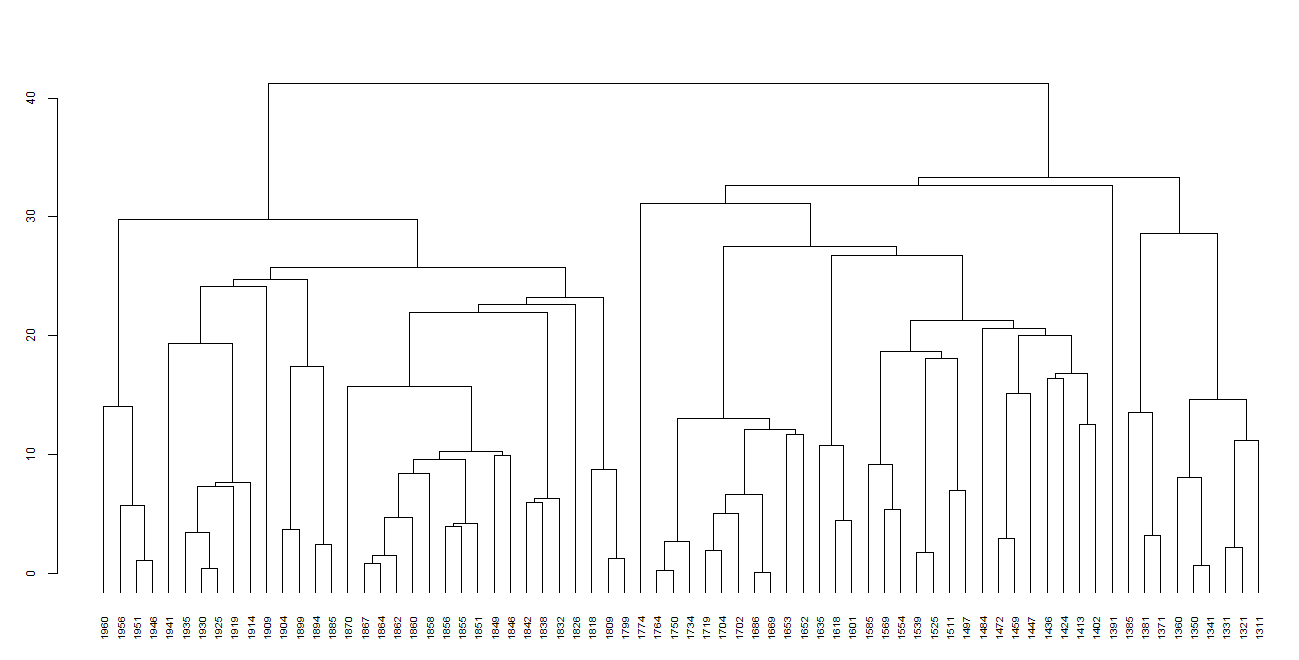


**Figure S13**. Dendrogram of the CONISS analysis of the paleoelementome of Lake Azul.
